# AVD Annals of Vascular Diseases

**DOI:** 10.3400/avd.cv.20-01000

**Published:** 2020-12-25

## No. 1

### EDITORIAL

**1 Flexions of the Popliteal Artery and the Culture Could Challenge the Outcomes of the Endovascular Procedures**

Rei Ukita, PhD and José Antonio Diaz, MD

### REVIEW ARTICLES

Therapeutic Angiogenes Update

**4 Therapeutic Arteriogenesis/Angiogenesis for Peripheral Arterial Disease by Nanoparticle-Mediated Delivery of Pitavastatin into Vascular Endothelial Cells**

Takuya Matsumoto, MD, PhD, FACS, Sho Yamashita, MD, Shinichiro Yoshino, MD, Shun Kurose, MD, Koichi Morisaki, MD, PhD, Kaku Nakano, PhD, Jun-ichiro Koga, MD, PhD, Tadashi Furuyama, MD, PhD, Masaki Mori, MD, PhD, FACS, and Kensuke Egashira, MD, PhD, FAHA, FESC

**13 Impact of Therapeutic Angiogenesis Using Autologous Bone Marrow-derived Mononuclear Cell Implantation in Patients with No-option Critical Limb Ischemia**

Kenji Yanishi, MD, PhD, Keisuke Shoji, MD, Ayumu Fujioka, MD, Yusuke Hori, MD, Arito Yukawa, and Satoaki Matoba, MD, PhD

Updates on Image Diagnosis in Aortic Disease

**23 Updates on Computed Tomography Imaging in Aortic Aneurysms and Dissection**

Ryoichi Tanaka, MD, PhD, Kunihiro Yoshioka, MD, PhD, and Akihiko Abiko, MD, PhD

### REVIEW ARTICLES

**28 The Role of Carotid Stump Pressure in Carotid Endarterectomy: A Systematic Review and Meta-Analysis**

Ali Kordzadeh, MBBS, MSc, MD, VA-BC, FEBS, FEBVS, Omar Ahmed Abbassi, MBBS, Ioannis Prionidis, PhD, FRCS, and Emad Shawish, MBBS, MSc, FEBS, FEBVS

**38 Recommendations for VTE Prophylaxis in Medically Ill Patients**

Nedaa Skeik, MD, FACC, FACP, FSVM, RPVI and Emily Westergard, DO

### ORIGINAL ARTICLES

**45 Acute Kidney Injury Following Elective Open Aortic Repair with Suprarenal Clamping**

Nobu Yokoyama, MD, Takao Nonaka, MD, Naoyuki Kimura, MD, PhD, Yusuke Sasabuchi, MD, MPH, PhD, Daijiro Hori, MD, PhD, Wataru Matsunaga, MD, Tomonari Fujimori, MD, Kosuke Miyoshi, MD, Harunobu Matsumoto, MD, PhD, and Atsushi Yamaguchi, MD, PhD

**52 Development of a Web Application That Evaluates Suture Performance in Off-the-Job Training**

Kazuhiro Miyahara, MD, Katsuyuki Hoshina, MD, PhD, Takafumi Akai, MD, PhD, Toshihiko Isaji, MD, PhD, Kota Yamamoto, MD, PhD, and Toshio Takayama, MD, PhD

**56 Duration from Wound Occurrence to Referral to a Vascular Center in Japanese Patients with Critical Limb Ischemia**

Mitsuyoshi Takahara, MD, PhD, Osamu Iida, MD, Yoshimitsu Soga, MD, PhD, Akio Kodama, MD, PhD, Hiroto Terashi, MD, PhD, Makoto Utsunomiya, MD, PhD, Jin Okazaki, MD, PhD, Nobuyoshi Azuma, MD, PhD, for the SPINACH study investigators

**63 Predictors of Poor Quality of Life after Primary Lower Limb Deep Venous Thrombosis: A Perspective from a Developing Nation**

Nadeem Ahmad Siddiqui, MBBS, FCPS, Muhammad Asad Moosa, MBBS, FCPS, Fareed Ahmad Shaikh, MBBS, FCPS, Noman Shahzad, MBBS, FCPS, Shahid Nazir, MBBS, and Ziad Sophie, MD

### CASE REPORTS

**69 Mobile Thrombus in the Ascending Aorta**

Yujiro Kawai, PhD, Kiyoshi Koizumi, MD, PhD, Takahito Itoh, PhD, Minami Iio, PhD, and Hideyuki Shimizu, MD, PhD

**72 Stent-Graft Relining by Combined Aortic Cuff with Double-D Technique for Type IIIb or V Endoleak after Endovascular Aneurysm Repair: A Case Report**

Takaaki Maruhashi, MD, Hiroshi Nishimaki, MD, PhD, Yukihisa Ogawa, MD, PhD, Kiyoshi Chiba, MD, PhD, Akiyuki Kotoku, MD, PhD, Yuka Sakurai, MD, and Takeshi Miyairi, MD, PhD

**76 Hybrid Repair for Mega-Aortic Syndrome due to Giant Cell Aortitis in a Heart Failure Patient**

Takehiro Inoue, MD, Kosuke Fujii, MD, Shigeo Kino, MD, Shintaro Yukami, MD, Naoya Miyashita, MD, and Hitoshi Kitayama, MD

**81 Successful Endovascular Repair for Abdominal Aortic Aneurysm Presenting as Aortoduodenal Syndrome**

Yasuhiko Kawaguchi, MD, Yasuhiro Oba, MD, Yoriyasu Suzuki, MD, Mototsugu Tamaki, MD, Yutaka Koyama, MD, and Hideki Kitamura, MD, PhD

**86 A Case of Chronic Limb-Threatening Ischemia with Heel Ulcers Cured by Revascularization and Partial Calcanectomy**

Takamitsu Tatsukawa, MD, Shinsuke Kikuchi, MD, PhD, Ai Tochikubo, MD, Seima Ohira, MD, Ranko Hinooka, RN, Daiki Uchida, MD, PhD, Satomi Abe, MD, PhD, Tetsuo Ota, MD, PhD, and Nobuyoshi Azuma, MD, PhD

**90 Recurrence of Aortoenteric Fistula after Endovascular Aortic Repair**

Daisuke Arima, MD, Yoshihiro Suematsu, MD, PhD, Kanan Kurahashi, MD, Takaharu Shimizu, MD, Satoshi Nishi, MD, and Akihiro Yoshimoto, MD

**93 Cough, Aortobronchial Fistula, and Air Migration in the Remnant Aneurysm Sac: An Unforeseen Path?**

Kilsoo Yie, MD

**96 Pulmonary Embolectomy for Acute Pulmonary Embolism: A Word of Caution**

Tomohiko Inui, MD, PhD, Keiichi Ishida, MD, PhD, Hiroki Kohno, MD, PhD, Kaoru Matsuura, MD, PhD, Hideki Ueda, MD, PhD, Yusaku Tamura, MD, PhD, Michiko Watanabe, MD, PhD, Yuichi Inage, MD, PhD, Yasunori Yakita, MD, and Goro Matsumiya, MD, PhD

**100 Organ Protection for Radical Debridement to Prevent Recurrence of Infection after Zone 1 Thoracic Endovascular Aortic Aneurysm Repair: A Case Report**

Yojiro Koda, MD, Hirohisa Murakami, MD, PhD, Hitoshi Matsuda, MD, PhD, and Nobuhiko Mukohara, MD, PhD

**103 Zone 0 Thoracic Endovascular Aortic Repair Using Reverse Extra-Anatomical Aortic Arch Debranching Technique for an Anastomotic Pseudoaneurysm and Acute Aortic Dissection that Developed after Bentall’s Surgery Combined with Sjögren’s Syndrome**

Daisuke Arima, MD, Yoshihiro Suematsu, MD, PhD, Satoshi Nishi, MD, Kanan Kurahashi, MD, Takaharu Shimizu, MD, and Akihiro Yoshimoto, MD

## No. 2

### EDITORIAL

**107 Introduction**

Juno Deguchi, MD, PhD

### COMMENTARY

**108 Does It Matter? Commentary on “Prosthetic Graft Dilation at the Aortic Arch in the Era of Hybrid Aortic Surgery”**

Yutaka Okita, MD, PhD

### REVIEW ARTICLES

Therapeutic Angiogenes Update

**109 Therapeutic Angiogenesis Using HGF Plasmid**

Fumihiro Sanada, MD, PhD, Tatsuya Fujikawa, MS, Kana Shibata, Yoshiaki Taniyama, MD, PhD, Hiromi Rakugi, MD, PhD, and Ryuichi Morishita, MD, PhD

**116 Therapeutic Angiogenesis with Sound Waves**

Tomohiko Shindo, MD, PhD and Hiroaki Shimokawa, MD, PhD

Angiosome and Revascularization

**126 Angiosome～From the Standpoint of Bypass Surgery**

Juno Deguchi, MD, PhD

### REVIEW ARTICLE

**132 May–Thurner and Paget–Schroetter Syndromes: A Review**

Zia Ur Rehman, MBBS, FCPS, ChM Vascular and Endovascular Surgery

### ORIGINAL ARTICLES

**137 Mid-Term Results of Frozen Elephant Trunk Technique for Chronic Aortic Dissection**

Yoshitaka Yamane, MD, Keijiro Katayama, PhD, Tomokuni Furukawa, PhD, Haruna Shimizu, MD, Takanobu Okazaki, MD, Taiichi Takasaki, PhD, Tatsuya Kurosaki, PhD, and Shinya Takahashi, PhD

**144 The Comparison between Axillofemoral Bypass and Endovascular Treatment for Patients with Challenging Aortoiliac Occlusive Disease as Alternative Treatment to Aortofemoral Bypass**

Masato Nishizawa, MD, PhD, Kimihiro Igari, MD, PhD, Sotaro Katsui, MD, PhD, Toshifumi Kudo, MD, PhD, and Hiroyuki Uetake, MD, PhD

**151 The Role of Syk in Inflammatory Response of Human Abdominal Aortic Aneurysm Tissue**

Ryo Kanamoto, MD, Hiroki Aoki, MD, PhD, Aya Furusho, MD, PhD, Hiroyuki Otsuka, MD, PhD, Yusuke Shintani, MD, PhD, Satoru Tobinaga, MD, PhD, Shinichi Hiromatsu, MD, PhD, Yoshihiro Fukumoto, MD, PhD, and Hiroyuki Tanaka, MD, PhD

**158 Peripheral Arterial Injuries in Children: An Audit at a University Hospital in Developing Country**

Zia Ur Rehman, MBBS, FCPS, Amna Riaz, MBBS, and Zafar Nazir, MBBS, FRCS

**163 Prosthetic Graft Dilation at the Aortic Arch in the Era of Hybrid Aortic Surgery**

Daijiro Hori, MD, PhD, Sho Kusadokoro, MD, Toshikazu Shimizu, MD, Naoyuki Kimura, MD, PhD, and Atsushi Yamaguchi, MD, PhD

**170 Grip Exercise of Non-Paretic Hand Can Improve Venous Return in the Paretic Arm in Stroke Patients: An Experimental Study in the Supine and Sitting Positions**

Hiroyuki Hayashi, OTR, PhD and Motoyuki Abe, MD, PhD

### CASE REPORTS

**176 Spinal Cord Infarction after Transcatheter Embolization of Pelvic Arteriovenous Malformation**

Shinji Wada, MD, Yukihisa Ogawa, MD, PhD, Kotaro Hosoi, MD, Kazuki Hashimoto, MD, Junji Moriya, MD, PhD, Shingo Hamaguchi, MD, Hidemichi Ito, MD, PhD, Homare Nakamura, MD, PhD, and Hidefumi Mimura, MD

**180 Parallel Placement of Excluder Legs for the Treatment of a Type IIIb Endoleak Using AFX2**

Hiroaki Kato, MD, Noriyuki Kato, MD, Ken Nakajima, MD, Takatoshi Higashigawa, MD, Takafumi Ouchi, MD, Shuji Chino, MD, and Hajime Sakuma, MD

**183 Emergency Endovascular Aneurysm Repair Coupled with Staged Omentopexy for Primary Aorto-Duodenal Fistula**

Shuhei Miura, MD, Yoshihiko Kurimoto, MD, PhD, Kosuke Ujihira, MD, Takahiko Masuda, MD, Yohsuke Yanase, MD, PhD, Yutaka Iba, MD, PhD, Ryushi Maruyama, MD, PhD, and Akira Yamada, MD, PhD

**187 Spontaneous Collapse of Balloon Expandable Stent in Heavily Calcified Aorta**

Taehwan Kim, MD, Shigeo Ichihashi, MD, Amane Kozuki, MD, Daichi Fujimoto, MD, Satoru Nagatomi, MD, Francesco Bolstad, MD, Junya Shite, MD, and Kimihiko Kichikawa, MD

**191 Hybrid Repair of Kommerell’s Diverticulum with Embolization of Aberrant Left Subclavian Artery**

Masamichi Matsumori, PhD, Motoharu Kawashima, MD, Yoshikatsu Nomura, PhD, Hirohisa Murakami, PhD, and Nobuhiko Mukohara, PhD

**194 Iatrogenic Iliac Vein Compression Syndrome Caused Because of Inappropriate Length and Positioning of Vascular Graft**

Yusuke Enta, MD, Akiko Tanaka, MD, Makoto Saigan, MD, Norio Tada, MD, PhD, and Masaki Hata, MD

**198 Popliteal Artery Entrapment Syndrome in Siblings**

Yuki Orimoto, MD, Hiroki Mitsuoka, MD, Yusuke Imaeda, MD, Takahiro Arima, MD, Yuki Maruyama, MD, Tetsuya Yamada, MD, and Hiroyuki Ishibashi, MD

**202 Peripheral Arterial Disease with Small Aorta Syndrome: A Case Report**

Noriyasu Masuda, MD, PhD and Kazuhiko Uwabe, MD, PhD

### ANNUAL REPORT

**205 2017 JAPAN Critical Limb Ischemia Database (JCLIMB) Annual Report**

The Japanese Society for Vascular Surgery JCLIMB Committee, NCD JCLIMB Analytical Team

## No. 3

### ORIGINAL ARTICLES

Selection from Japanese Journal of Vascular Surgery 2019

**235 Results of Stenting for Central Venous Occlusions and Stenoses in the Hemodialysis Patients**

Daihiko Eguchi, MD, PhD and Kenichi Honma, MD, PhD

**240 Predictive Factor of the Possibility for Aortic Side Branches Coil Embolization during Endovascular Abdominal Aortic Aneurysm Repair**

Atsushi Aoki, MD, PhD, Kazuto Maruta, MD, PhD, Norifumi Hosaka, MD, PhD, Tomoaki Masuda, MD, PhD, Tadashi Omoto, MD, PhD, and Yui Horikawa, MD

Selection from the Journal of Japanese College of Angiology 2019

**248 Neomedia Repair of the Valsalva Sinus in the Treatment of Acute Type-A Aortic Dissection: Long-term Effectiveness and a Case of Pathology**

Nobuhisa Ohno, MD, PhD, Toshi Maeda, MD, Otohime Kato, MD, Hirofumi Sato, MD, Go Ueno, MD, and Kosuke Yoshizawa, MD

### ORIGINAL ARTICLES

**255 A Retrospective Study of Deep Vein Insufficiency Treatment Device: ICT**

Turhan Yavuz, Prof. MD, Altay Nihat Acar, MD, Kubra Yavuz, MD, and Evren Ekingen, MD

**261 Preoperative Neck Angulation is Associated with Aneurysm Sac Growth Due to Persistent Type Ia Endoleak after Endovascular Abdominal Aortic Aneurysm Repair**

Yoshimasa Seike, MD, Tetsuya Fukuda, MD, PhD, Koki Yokawa, MD, Yosuke Inoue, MD, Takayuki Shijo, MD, Kyokun Uehara, MD, PhD, Hiroaki Sasaki, MD, PhD, and Hitoshi Matsuda, MD, PhD

**269 Clinical Utility of Coil in Plug Method (CIP) for Internal Iliac Artery Embolization during Endovascular Aortic Aneurysm Repair**

Akiyuki Kotoku, MD, PhD, Yukihisa Ogawa, MD, PhD, Kiyoshi Chiba, MD, PhD, Takaaki Maruhashi, MD, Hidefumi Mimura, MD, PhD, Takeshi Miyairi, MD, PhD, and Hiroshi Nishimaki, MD, PhD

**273 Effect of Atheromatous Aorta on Thromboembolic Complications after Endovascular Aortic Aneurysm**

Tsunehiro Shintani, MD, PhD, Hiroshi Mitsuoka, MD, PhD, Yuto Hasegawa, MD, Masanori Hayashi, MD, Kayoko Natsume, MD, Kazuhiro Ookura, MD, Yasunori Sato, PhD, and Hideaki Obara, MD, PhD

**281 Results of Surgical Repair of Hilar Renal Artery Aneurysm to Preserve Renal Blood Flow**

Takumi Kawase, MD, Yosuke Inoue, MD, Jiro Matsuo, MD, Atsushi Omura, MD, PhD, Yoshimasa Seike, MD, Kyokun Uehara, MD, PhD, Hiroaki Sasaki, MD, PhD, and Hitoshi Matsuda, MD, PhD

**286 Tension-Free Management of Surgical Wound in Paramalleolar Bypass**

Yoshihiko Tsuji, MD, Ikuro Kitano, MD, and Yoriko Tsuji, MD

**291 Open-Label Multicenter Registry on the Outcomes of In-Stent Restenosis Treated by Balloon Angioplasty with Optical Frequency Domain Imaging in the Superficial Femoral Artery (ISLAND-SFA Study)**

Kenichi Yanaka, MD, PhD, Akihide Konishi, MD, PhD, Toshiro Shinke, MD, PhD, Amane Kozuki, MD, PhD, Hiroyuki Kawamori, MD, PhD, Yoshiro Tsukiyama, MD, PhD, Osamu Iida, MD, Makoto Kadotani, MD, PhD, Takashi Omori, PhD, and Ken-ichi Hirata, MD, PhD

**300 Heterogeneity of Age and Its Associated Features in Patients with Critical Limb Ischemia**

Mitsuyoshi Takahara, MD, PhD, Osamu Iida, MD, Yoshimitsu Soga, MD, PhD, Akio Kodama, MD, PhD, Hiroto Terashi, MD, PhD, Kenji Suzuki, MD, Ikuo Sugimoto, MD, PhD, and Nobuyoshi Azuma, MD, PhD

### CASE REPORTS

**308 A Case of Collapsed Stent Graft, Severe Lower Limb Ischemia, and Ruptured Abdominal Aortic Aneurysm Due to Type B Acute Aortic Dissection 3 Years after Endovascular Aneurysm Repair**

Yusuke Motoji, MD, Takayoshi Kato, MD, Jun Seki, MD, Kosuke Tsumura, MD, Shinji Tomita, MD, PhD, and Yasuhide Okawa, MD

**312 A Case of Periaortic Lymphoma Mimicking Complicated Type B Acute Aortic Dissection: A Pitfall in the Endovascular Surgery Era**

Yuki Imamura, MD, Norihiro Kondo, MD, PhD, Yoshiaki Saito, MD, PhD, Kaoru Ogawa, MD, Mari Chiyoya, MD, PhD, and Ikuo Fukuda, MD, PhD

**316 Thoracic Endovascular Aortic Repair for Ruptured Intercostal Patch Aneurysm Following Thoracoabdominal Aortic Aneurysm Repair**

Soichiro Henmi, MD, PhD, Hidekazu Nakai, MD, Katsuhiro Yamanaka, MD, PhD, Atsushi Omura, MD, PhD, Takeshi Inoue, MD, and Kenji Okada, MD, PhD

**319 Parallel Placement of Excluder Legs to Treat Abdominal Aortic Aneurysms with Aortoiliac Occlusive Lesion**

Hiroaki Kato, MD, Noriyuki Kato, MD, Ken Nakajima, MD, Takatoshi Higashigawa, MD, Takafumi Ouchi, MD, Shuji Chino, MD, Toshiya Tokui, MD, and Hajime Sakuma, MD

**322 A Case of Total Excision of a Thrombosed-Venous Aneurysm in the Sural Vein**

Masato Nishizawa, MD, PhD, Kimihiro Igari, MD, PhD, Masayuki Hirokawa, MD, PhD, Nobuhisa Kurihara, MD, PhD, Sotaro Katsui, MD, PhD, Toshifumi Kudo, MD, PhD, and Hiroyuki Uetake, MD, PhD

**326 Therapeutic Strategies for Infectious Multiple Aortic Aneurysms: Thoracoabdominal Aortic Aneurysm Treatment Using a Fenestrated Stent-Graft**

Satoshi Okugi, MD, Takashi Azuma, MD, Yoshihiko Yokoi, MD, Satoru Domoto, MD, PhD, and Hiroshi Niinami, MD, PhD

**330 Understanding Vascular Anatomy is Key to Successful Endovascular Treatment of Pancreaticoduodenal Artery Aneurysms**

Koji Hirano, MD, Toshiya Tokui, MD, Bun Nakamura, MD, Ryosai Inoue, MD, Reina Hirano, MD, Yasumi Maze, MD, Shuji Chino, MD, Hisato Ito, MD, Yu Shomura, MD, and Motoshi Takao, MD

**335 Spinal Cord Ischemia Following Endovascular Repair of Infrarenal Abdominal Aortic Aneurysm**

Shingo Nakai, MD, Tetsuro Uchida, MD, PhD, Yoshinori Kuroda, MD, PhD, Atsushi Yamashita, MD, Eiichi Oba, MD, PhD, Kimihiro Kobayashi, MD, Tomonori Ochiai, MD, and Mitsuaki Sadahiro, MD, PhD

**339 Endovascular Aortic Repair under Extracorporeal Cardiac Support in a Patient with an Abdominal Aortic Aneurysm Impending Rupture and Aortic Stenosis: A Case Report**

Yohei Kawatani, MD, PhD, Motoshige Yamasaki, MD, PhD, and Atsushi Oguri, MD, PhD

**343 Rapidly Progressed Distal Arch Aneurysm with Distal Open Stent Graft-Induced New Entry Caused by “Spring-Back” Force**

Masami Takagaki, MD, PhD, Hirofumi Midorikawa, MD, PhD, Hiroki Yamaguchi, MD, PhD, Hiromasa Nakamura, MD, PhD, Tasuku Kadowaki, MD, Yousuke Ueno, MD, Takaki Uchida, MD, and Tomoyuki Aoki, MD

**347 Total Percutaneous Femoral Approach for Branched Custom-Made Device Endovascular Repair of Thoracoabdominal Aneurysm**

Benjamin Dak Keung Leong, FRCS and Feona Sibangun Joseph, MS

**351 Long-Term Survival in a Case of Primary Leiomyosarcoma of the Inferior Vena Cava in Which PET-CT Follow-up Was Useful**

Masahiro Aiba, MD, PhD and Ikutaro Kigawa, MD, PhD

**355 Surgical Management of Aortoenteric Erosion Due to Pulsatile Stress After Aneurysm Repair: A Case Report**

Shingo Kunioka, MD, Hiroto Kitahara, MD, Seima Ohira, MD, Yuki Tada, MD, Nobuyuki Akasaka, MD, and Hiroyuki Kamiya, MD

## No. 4

### REVIEW ARTICLE

**359 Nosological Consideration of Arterial Aneurysms Associated with Klippel–Trenaunay Syndrome**

Takashi Ohta, MD and Shinobu Matsubara, MD

### ORIGINAL ARTICLES

**365 Surgical Excision of Carotid Body Tumor at an Early Stage Has Best Outcome: Result of 22 Cases along with Literature Review**

Rashid Usman, MBBS, MRCS, FCPS, FACS, FRCS, Muhammad Jamil, MBBS, FCPS, MRCS, OJT Vascular, FACS, FRCS, and Aaiza Aman, MBBS

**370 Administration of Direct Oral Anticoagulant Immediately after Unfractionated Heparin Bolus for the Treatment of Intermediate–High-Risk Pulmonary Thromboembolism**

Nobuhiro Hara, MD, Keita Watanabe, MD, Ryoichi Miyazaki, MD, Tomofumi Nakamura, MD, Tetsumin Lee, MD, Yasutoshi Nagata, MD, Toshihiro Nozato, MD, Takamichi Miyamoto, MD, Toru Obayashi, MD, and Takashi Ashikaga, MD

**377 Effect of Endovascular Treatment on Systemic Vascular Resistance in Patients with Lower-Limb Peripheral Artery Disease**

Hidetsugu Nomoto, MD, Toshihiro Nozato, MD, Shu Yamashita, MD, Masahito Suzuki, MD, Tomoyo Sugiyama, MD, Tetsuo Oumi, MD, Masakazu Ohno, MD, Shigeo Shimizu, MD, Takashi Ashikaga, MD, and Yasuhiro Satoh, MD

**384 Long-Term Clinical Outcomes of Thoracic Endovascular Aortic Repair for Arch Aneurysms with the Najuta Thoracic Stent-Graft System**

Hiroshi Sato, MD, PhD, Joji Fukada, MD, PhD, Yukihiko Tamiya, MD, PhD, Takuma Mikami, MD, Tsuyoshi Sibata, MD, Ryo Harada, MD, PhD, Syuichi Naraoka, MD, PhD, Takeshi Kamada, MD, PhD, Nobuyoshi Kawaharada, MD, PhD, and Yoshihiko Kurimoto, MD, PhD

**390 The Cleaner XT™ Device as an Endovascular Adjunct for Pharmacomechanical Thrombolysis of Thrombosed Arteriovenous Fistulas and Grafts**

Khian Wan Sarah Joy Huan, MBBS (Singapore), MRCS(Ed), Chieh Suai Tan, MBBS (Singapore), MRCP (UK), Deborah Chua, MB BCh BAO (Hons), Charyl Jia Qi Yap, BSc, Ru Yu Tan, MBBS (Malaysia), MRCP (UK), Tze Tec Chong, MBBS, FACS, RPVI, and Tjun Yip Tang, MB BChir, MA (Cantab), FRCS (Glas), FRCS (Eng), MD (Vascular Surg, Cambridge), FAMS

**397 Noninvasive Technique for Measuring Central Venous and Arterial Pressure Using Controlled Compression Sonography**

Hiroshi Tomoeda, MD, PhD, Kentaro Sawada, MD, PhD, and Shingo Chihara, MD, PhD

**404 Salvage of Infected Prosthetic Grafts at the Groin or Thigh Using Muscle Flap Coverage**

Kiyoshi Tanaka, MD, PhD, Shinsuke Mii, MD, PhD, Masaru Ishida, MD, PhD, Atsushi Guntani, MD, PhD, Eisuke Kawakubo, MD, PhD, Shinichi Tanaka, MD, PhD, Ryosuke Yoshiga, MD, and Jin Okazaki, MD, PhD

**410 Interface Pressures Derived from a Tubular Elastic Bandage**

Kotaro Suehiro, MD, Noriyasu Morikage, MD, Takasuke Harada, MD, Makoto Samura, MD, Takashi Nagase, MD, Yuriko Takeuchi, MD, Takahiro Mizoguchi, MD, Ryo Suzuki, MD, Hiroshi Kurazumi, MD, and Kimikazu Hamano, MD

### CASE REPORTS

**414 Successful Two-Stage Treatment for Coarctation of the Aorta-postductal Type and Aortic Regurgitation with Thoracic Endovascular Aortic Repair and Aortic Valve Replacement**

Ryuta Seguchi, MD, PhD, Takafumi Horikawa, MD, Ryuta Kiuchi, MD, PhD, Junichiro Sanada, MD, PhD, Hiroshi Ohtake, MD, PhD, and Go Watanabe, MD, PhD

**418 Peripheral Inflammatory Superior Mesenteric Artery Aneurysm Diagnosed by Intraoperative and Histological Findings: A Case Report**

Yasuo Suehiro, MD, PhD, Hiroyuki Seo, MD, Yuko Kubota, MD, PhD, Shigefumi Suehiro, MD, PhD, and Hidekazu Hirai, MD, PhD

**422 Simultaneous Aortic Valve-in-Valve and Ascending Stent Grafting for Prosthetic Valve Stenosis and Ascending Flap**

Yoshiyuki Yamashita, MD, PhD, Kazuo Shimamura, MD, PhD, Koichi Maeda, MD, PhD, Yu Yamada, MD, Toru Ide, MD, Toru Kuratani, MD, PhD, and Yoshiki Sawa, MD, PhD

**426 A Case of Circumferential Type A Aortic Dissection with Intimo–Intimal Intussusception**

Yoichi Yamashita, MD, PhD, Sayako Nakagawa, MD, Shohei Kitamoto, MD, Kosuke Sakamoto, MD, and Taiko Horii, MD, PhD

**430 Open Surgical Repair for a Giant Common Hepatic Artery Aneurysm with Difficulty in Proximal Arterial Clamping: A Case Report**

Naoki Asano, MD, Takashi Yamauchi, MD, PhD, Kazunori Ota, MD, Kazuho Niimi, MD, Masahito Saito, MD, PhD, Shigeyoshi Gon, MD, PhD, Kei Torikai, MD, PhD, Shinichi Ban, MD, PhD, and Hiroshi Takano, MD, PhD

**434 Endovascular Repair for Abdominal Aortic Rupture Caused by Periaortic Mantle Cell Lymphoma**

Yukihiro Matsuno, MD, PhD, Shohei Mitta, MD, Yukio Umeda, MD, PhD, Ryota Watanabe, MD, and Yoshio Mori, MD, PhD

**437 Inferior Vena Cava Filter-Induced Thrombosis: Filter Insertion Prior to Catheter-Directed Thrombolysis and Successful Double-Filter Retrieval after Prolonged Indwelling Time**

Wakako Fukuda, MD, PhD, Takashi Shibuya, MD, PhD, Kenichi Watanabe, MD, PhD, Masato Ohno, MD, Tomoaki Kudo, MD, PhD, Ikuo Fukuda, MD, PhD, and Mitsunori Kaneko, MD, PhD

**441 Emergent Transcatheter Arterial Embolization via a Transpopliteal Approach for Internal Iliac Artery Injury during Lumbar Disk Surgery in the Prone Position**

Kiyoshi Chiba, MD, Yukihisa Ogawa, MD, Kenji Murakami, MD, Shota Kita, MD, Hirotoshi Suzuki, MD, Masahide Komagamine, MD, Kan Nawata, MD, PhD, Masahide Chikada, MD, PhD, Hiroshi Nishimaki, MD, PhD, and Takeshi Miyairi, MD, PhD

**444 Compression of the Left Atrium and Left Ventricle by a Thoracoabdominal Aortic Aneurysm: A Case Report**

Koichi Nagaya, MD, PhD, Masaaki Naganuma, MD, PhD, Yasushi Kudoh, MD, Nobuaki Suzuki, MD, and Shinya Masuda, MD, PhD

**447 A Case of Superior Mesenteric Venous Thrombosis Managed by Thrombectomy without Bowel Resection**

Wakiko Hiranuma, MD, Takuya Shimizu, MD, PhD, Miki Takeda, MD, Takayuki Matsuoka, MD, PhD, Tadanori Minagawa, MD, PhD, Toshiaki Fukutomi, MD, PhD, Masato Ohara, MD, PhD, and Shunsuke Kawamoto, MD, PhD

**450 Successful Repair of Superior Mesenteric Artery Aneurysm with Reconstruction of Branches**

Ryota Matsumoto, MD, Takashi Shibuya, MD, PhD, Fumiyoshi Saijo, MD, Kenichi Watanabe, MD, PhD, and Yoshiki Sawa, MD, PhD

**454 Endovascular Treatment of a Ruptured Pseudoaneurysm of the Intercostal Patch after Descending Aortic Aneurysm Repair**

Yoshikatsu Nomura, MD, PhD, Ryota Kawasaki, MD, PhD, Motoharu Kawashima, MD, Hiroshi Tanaka, MD, PhD, and Hirohisa Murakami, MD, PhD

**457 Intraoperative Findings of an Atypical Type II Endoleak from an Artery within the Aneurysmal Wall after Endovascular Aneurysm Repair**

Baku Takahashi, MD, Shinji Kamiya, MD, Kengo Ohta, MD, Yoshiharu Mori, MD, Toshiyuki Yamada, MD, PhD, Yosuke Nakai, MD, Hisao Suda, MD, PhD, and Akira Mishima, MD, PhD

**461 Endovenous Thermal Ablation for a Varicose Vein Patient with Factor XII Deficiency: A Case Report**

Nozomu Shirasugi, MD, PhD, Sadaaki Horiguchi, MD, PhD, Takamitsu Tanaka, MD, PhD, Hiroyuki Shirato, MD, PhD, Hisako Ono, AS, and Kazuo Kawasugi, MD, PhD

**465 Successful Embolectomy of the Plantar Artery Occlusion Due to Thromboembolism**

Kazunori Hashimoto, MD, Harunobu Matsumoto, MD, PhD, Takao Nonaka, MD, Naoyuki Kimura, MD, PhD, Koichi Yuri, MD, PhD, and Atsushi Yamaguchi, MD, PhD

**469 Hepatic Vein Aneurysm: An Extremely Rare Case with Successful Embolization**

Surasit Akkakrisee, MD, Keerati Hongsakul, MD, and Tortrakoon Thongkan, MD

### ANNUAL REPORTS

**474 Vascular Surgery in Japan: 2014 Annual Report by the Japanese Society for Vascular Surgery**

The Japanese Society for Vascular Surgery Database Management Committee Member, and NCD Vascular Surgery Data Analysis Team

**494 Reviewers Index**

**496 Authors Index**

**500 Contents of Volume 13, 2020**

**509 Best Cited Articles 2020**

